# Pacemaking in cardiac tissue. From IK2 to a coupled‐clock system

**DOI:** 10.14814/phy2.13862

**Published:** 2019-01-03

**Authors:** Edward Carmeliet

**Affiliations:** ^1^ Katholieke Universiteit Leuven Leuven Belgium

**Keywords:** Biological pacemaker, conduction, ionic theory, patch

## Abstract

Initially, diastolic depolarization in Purkinje fibers was explained by deactivation of gK2 in the presence of inward current. Weakness of the hypothesis was a too negative reversal potential, sensitivity to external Na^+^ ions, existence of K^+^ depletion, and fake current during hyperpolarizing clamps. The development of a sinus node preparation of almost microscopic dimensions allowing uniform voltage clamps created new possibilities. Three different groups discovered in this improved node preparation an hyperpolarization induced time‐dependent inward current, with a reversal potential positive to the resting potential, carried by a mixture of Na^+^ and K^+^ ions. A new current, If, or funny current was born. It is not the only pacemaker current. The following sequence of currents (membrane clock) has been proposed: diastole starts as a consequence of IK deactivation and If activation; followed by activation of the T‐type Ca^2+^ current, Ca^2+^‐induced Ca^2+^ release from the SR, and activation of sodium‐calcium exchange current with further depolarization of the membrane till threshold of the L‐type Ca^2+^current is reached. The release of Ca^2+^ can also occur spontaneously independently from a T‐type Ca^2+^current. The system acts then as a primary intracellular clock. The review is completed by description of an evolution in the direction of biological pacing using induced pluripotent stem cells or transcription factors.

**See also:** https://doi.org/10.14814/phy2.13860 & https://doi.org/10.14814/phy2.13861

## The IK2 Hypothesis

Typical for the transmembrane electrical activity in the sinus and in Purkinje fibers is the existence of a slow diastolic depolarization. Different between the two tissues is their maximum diastolic potential and the upstroke at the end of the diastole. It is therefore not directly expected that the same current(s) will be found responsible for the two types of spontaneous depolarization.

**Figure 1:**
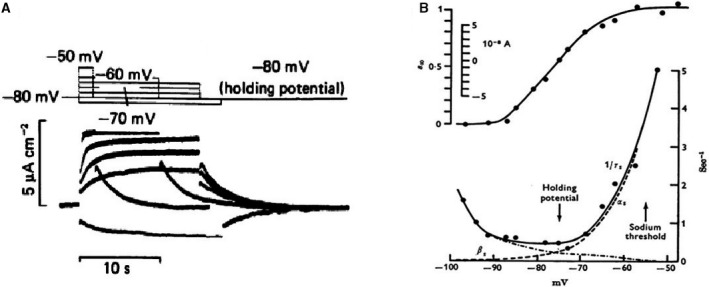
(A) Current changes in response to voltage clamp pulses within the pacemaker range of potentials. (B) Voltage dependence of IK2 kinetics. Top: Steady state activation curve. Bottom: time constants of activation and deactivation as a function of membrane potential (Noble and Tsien 1968). With permission.

**Figure 2:**
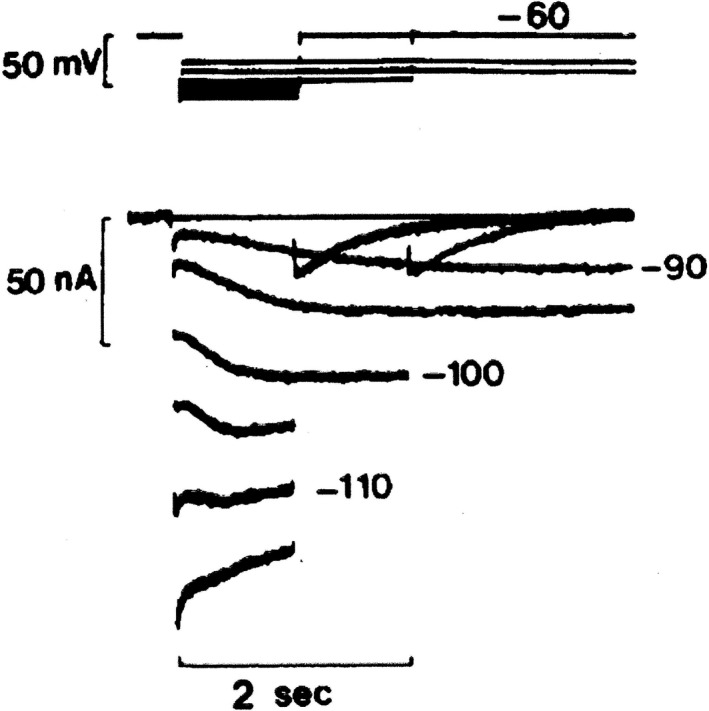
IK2 apparent reversal seen on hyperpolarization from a holding potential of −60 mV in a calf Purkinje fiber immersed in 3 mmol/L KCl, 140 mmol/L NaCl Tyrode solution. The apparent reversal occurs at about −110 mV. Notice the biphasic behavior at −110 mV (DiFrancesco 1985). With permission.

Three possible mechanisms to explain the process of diastolic depolarization in cardiac Purkinje fibers have been proposed by Weidmann (1956): (1) a decrease in K^+^ conductance consistent with the measured increase in impedance (2) an increase in Na^+^ conductance, (3) a decrease in Na^+^, K^+^‐pump activity. Notice that the last hypothetical possibility tacitly assumed an electrogenic Na^+^, K^+^‐pump. The first mechanism was called the gK‐decay hypothesis; it was selected by most electrophysiologists and has remained intact for more than 20 years. We will see later that a shift to the second hypothesis although in somewhat modified form (increase in Na^+^, K^+^current, the If current) was made in the early eighties. In the meantime, the gK‐decay hypothesis was taken up in the first mathematical model for the Purkinje fiber cardiac action potential (Noble 1962). In this model, two K^+^ conductances were assumed to play an important role: a time independent, inward‐going IK1 described experimentally for Purkinje fibers (Hutter and Noble 1960), (Carmeliet 1961) and a time‐dependent IK2 whose deactivation was responsible for the diastolic depolarization (see Noble (1975)). Later analysis (Noble and Tsien 1968) (Fig. 1) based on voltage clamp experiments in Purkinje fibers, demonstrated that the pacemaker current was activated between −90 and −60 mV, inward rectifying, and followed by deactivation on repolarization. In the presence of an inward background current, this would cause the diastolic depolarization. The gK‐decay hypothesis was in accord with the increase in slope resistance measured during diastole (Vassalle 1966). The current reversed at the *E*_K_ and this reversal potential changed with Ko as expected for a K^+^ electrode. The IK2 current was considered a pure K^+^ current. There were two problems however. (1) The reversal was some 5–10 mV more negative than the *E*_K_ estimated from the intracellular K^+^ concentration (Fig. 2). This deviation was explained by assuming a hyperactive Na^+^,K^+^‐pump which caused K^+^ depletion in the narrow extracellular clefts between the cells, (2) a second delicate aspect was the disappearance of IK2 current in Na^+^‐free medium, rather unexpected for a pure K^+^ current. These two weak points in the theory would continue to bother Denis Noble.

## Start of The (R)evolution: Fake Reversal Potentials and Better Preparations

How did the revolution start? What did provoke the change of mind? Two important evolutions: (1)The first was the accumulating evidence that application of voltage clamp hyperpolarizing pulses negative to the *E*_K_ was causing extracellular K^+^ concentration depletion in the narrow clefts between Purkinje fibers (Baumgarten and Isenberg 1977). The combination of a decreasing inward depletion current and the increasing time course of a real inward current possibly could create the false impression of a reversal potential (Fig. 2). (2) The second evolution was technical and consisted in the development of a sinus node preparation that could be used in voltage clamp experiments. Hopefully, this preparation was less subject to K+ accumulation‐depletion (less IK1?) than Purkinje fibers. The major discovery made with this new approach was the demonstration of an inward current activated upon hyperpolarization. Thanks to the technical skills of Hiroshi Irisawa and his group, a viable preparation of almost microscopic dimension (0.2 long and 0.3 mm in diameter) was developed from the rabbit SAN, a man‐made thin muscle strand. It could be used in the sucrose gap (Irisawa 1972a; Seyama 1976) or in the two‐microelectrode mode (Noma and Irisawa 1976). Afterwards Brown et al. (1977) developed a similar preparation from the frog sinus venosus. Voltage clamp experiments using the rabbit and frog preparations made it possible to discover an inward current system that was activated on hyperpolarization in a time‐dependent way. These findings were made by three different groups at practically the same time as I would like to illustrate in more detail. It was the end of the IK2 saga.

## Description of a New Current: Inward Current Activated on Hyperpolarization

### The Hiroshima group

The first attempt to measure voltage clamp currents in the newly developed sinoatrial node (SAN) preparation was made by Irisawa using the double sucrose gap method (Irisawa 1972b). I remember being present at the meeting in Brussels where Irisawa presented his man‐made sinoatrial preparation and I was very impressed. According to Irisawa himself (Irisawa et al. 1993) the importance of this report was limited; and I quote him: “the data were useful but far from ideal.” The reason for his not too enthusiastic reaction was the deficient voltage control because of large leakage problems using the small preparation in the two sucrose gap formula. Later, a preference was given to the two‐microelectrode approach, a much better solution. In the meantime, the preparation was further reduced to shortened specimens of 0.2–0.3 mm diameter.

**Figure 3:**
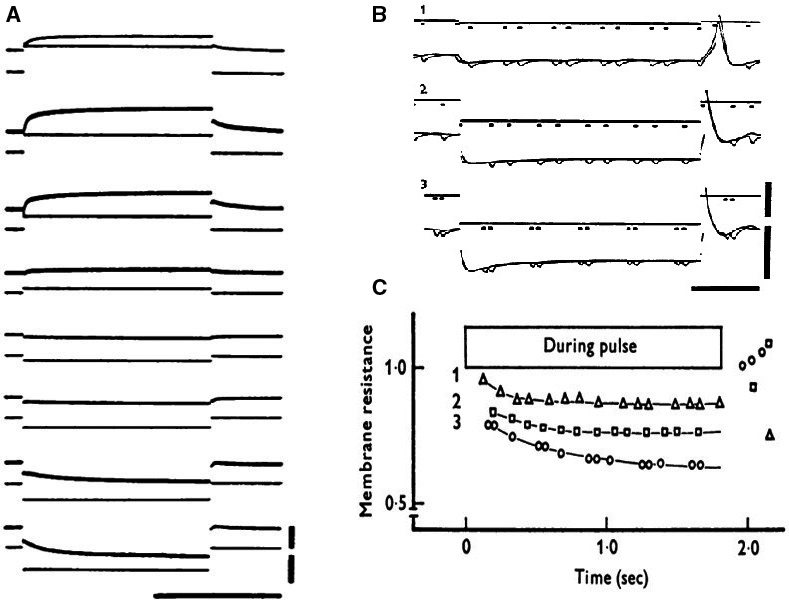
(A) Voltage clamp experiment; single sucrose, rabbit SAN preparation. Holding potential: −40 mV. Original records. Upper vertical bar indicates 1 × 10^−5^A and lower vertical bar 50 mV. Horizontal bar means 1 sec. In the uppermost record, current calibration should be read as 2 × 10^−5 ^A. (B): Changes in membrane resistances during application of anodal current. Upper vertical bar indicates 1 × 10^−5^ A and lower bar 50 mV. Horizontal bar shows 500 msec. (C): time course of changes in relative membrane resistance during application of anodal current (Seyama 1976). With permission.

**Figure 4:**
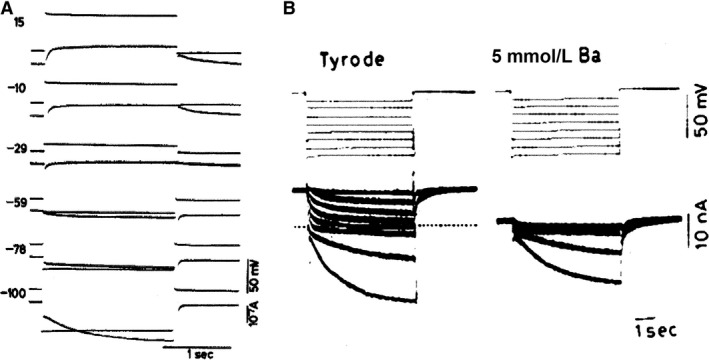
(A) Voltage clamp experiment on rabbit SAN (two microelectrodes)(Noma and Irisawa 1976). Membrane currents during depolarizing and hyperpolarizing voltage clamps. Holding potential:−40 mV. Displacement of membrane potential is indicated in mV at the left side of each record. (B) (Yanagihara and Irisawa 1980) Ba^2+^ions block IK without affecting If. Holding potential −10 mV. 5 s pulses were applied between −21 and −81 mV. 5 mmol/L Ba^2+^: currents between −21 and −61 mV completely blocked, currents at −73 and −81 mV unaffected. Dotted line: zero current. With permission.

Using the sinoatrial preparation in the single sucrose‐one microelectrode version, (Seyama 1976) described for the first time the presence of an inward current activated on hyperpolarization(Fig. 3A). Applying constant current pulses under similar conditions, he could prove that the membrane conductance increased (Fig. 3B and C). In the discussion of his paper, Seyama concludes: “In the SAN cell, though the ionic species could not be detected for this inward current, the results obtained support the notion that there is a time‐dependent increase of conductance to some ions with an equilibrium potential close to or positive to the resting potential**.”** With the two‐microelectrode voltage clamp version (Noma and Irisawa 1976), in the same year and the same lab, confirmed the existence of the inward current (Fig. 4A). A more substantial report on the dissection in two distinct currents, deactivation of an outward K^+^ current and activation of an inward current, appeared in 1980s (Yanagihara and Irisawa 1980) (Fig. 4B). Although the Hiroshima group was the first to describe the activation of an inward current upon hyperpolarization, they were not convinced that the current played an important role in pacemaking (Yanagihara and Irisawa 1980). The reason was that the potential level of activation was too negative and in the best case, according to their analysis, could deliver depolarizing current for the initial 50 msec of the diastolic depolarization. They considered the current as a background current which kept the membrane of the sinus node at a depolarized level (later called the insulator role). In a first conclusion, Irisawa, Noma, and Seyama considered deactivation of the IK as the most plausible explanation for the diastolic depolarization, but when it was shown that the outward current could be blocked by Ba^2+^ and pacemaking still continued, diastolic depolarization was explained as caused by activation of the Ca^2+^ current (Irisawa 1978; (Yanagihara and Irisawa 1980).

### The Oxford‐Calgary‐Milan group

**Figure 5:**
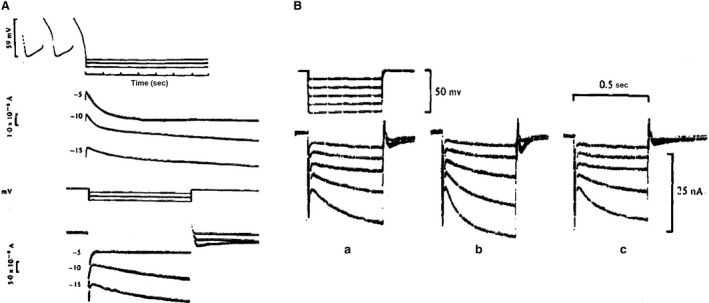
(A) Evidence for the existence of two currents on application of hyperpolarizing (Brown et al. 1977). Frog sinus venosus, double sucrose gap. Top: Voltage clamp applied at the end of an action potential and corresponding currents. At −5 mV: single current due to deactivation of iK; at −10 and −15 mV the current continued to drift downwards, suggesting the presence of two currents. Bottom: The same preparation was clamped at the resting potential and hyperpolarizing clamps applied: activation of an increasing inward current. (B) Rabbit sinoatrial node. Adrenaline effect on If. Hyperpolarizing voltage clamp pulses from holding potential −36 mV before (a), during (b) and after (c) perfusion with adrenaline 10^−7^M, TTX 10^−7^M and D600 10^−7^M (Brown et al. 1979b). With permission.

The next turn was for the Oxford group with voltage clamp experiments first on the frog sinus venosus (Brown et al. 1976, 1977). Figure 5A shows ionic currents during hyperpolarizing voltage steps from the maximum diastolic potential. They illustrate deactivation of an outward K^+^ current and at more negative potentials activation of an inward current. In Figure 5A (top), the clamp was preceded by an action potential during which an outward K^+^ current was activated. At 5 mV negative to the diastolic potential, deactivation of this K^+^ current is seen. At 10 and 15 mV negative to the maximum diastolic potential, the current continued to drift downwards throughout the hyperpolarizing clamps. This is not compatible with a simple deactivation of an outward current but suggests activation of an inward current. In Figure 5A (bottom), without preceding depolarization, deactivation of the outward current is supposedly absent and only the activation of the inward current is present.

These results, although in accord with the gK hypothesis, at the same time confirm the existence of a “queer inward current.” I cite the authors: “Another time‐dependent current system activated negative to the maximum diastolic potential appears to be present in the majority of the sinus preparations. Although the functional significance of the system is not clear, it is not an artefact of the double sucrose gap method, because a very similar current change had been reported by Seyama (1976) and by Noma and Irisawa (1976), using the single sucrose gap or the microelectrode techniques to voltage clamp rabbit sinoatrial node.” And the authors go on: “We ascribe this difficulty (measurement of reversal potential) to an additional conductance activated at more negative potentials than the maximum diastolic potential. The nature and function of this conductance *is obscure….”*

Progress was made when the If current was found to be increased by adrenaline, a substance well‐known for its accelerating action on the pacemaker(see Fig. 5B; Brown et al. 1979a). At the end of the Nature paper (Brown et al. 1979a), the authors ask the question about the nature of If and its comparison with IK2. Because of technical problems, they were unable to determine whether If had a reversal potential close to the equilibrium potential of K^+^ ions. They had no answer to the question whether or not “it could be equated with the current IK2 of the Purkinje fiber. Nevertheless, the resemblance to IK2 was striking: IK2 was deactivated by hyperpolarizations into the same voltage range as If and the change in If produced by adrenaline strongly resembles that produced in IK2 by a voltage shift of its kinetics.” *Conclusion*: If played a role in pacemaking.

### The Baltimore group

**Figure 6:**
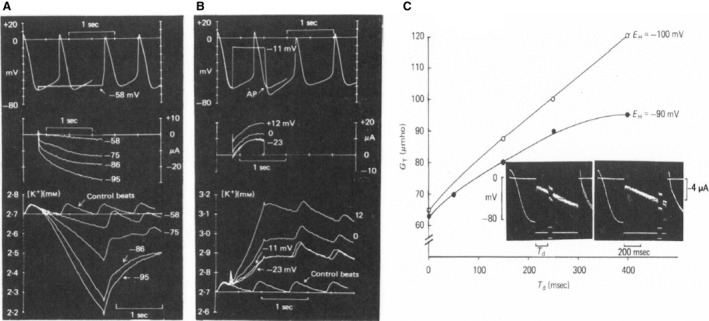
Potential dependence of K^+^ depletion (A) and accumulation (B) in SAN strips. Upper trace shows potential, middle trace clamp current, and bottom K^+^ concentration measured by a K^+^‐sensitive electrode. Depletion and accumulation are elicited by clamps initiated at the maximum diastolic potential. (C) Measurement of total membrane conductance during the “pacemaker” current. Plotted is the total membrane conductance measured at different times during activation of time‐dependent inward current at two holding potentials (−90 and −100 mV). The inset shows two records from the same experiment, demonstrating the small pulse perturbation technique used to measure the whole membrane conductance. Note that conductance increases during activation of the time‐dependent inward current. From Maylie et al. (1981); preliminary reports: Weiss et al. (1978) and Maylie et al. (1979). With permission.

The third group in competition was headed by Martin Morad, with the following three publications: (1) Weiss et al. (1978), (2) (Maylie et al. (1979), (3) Maylie et al. (1981). The first paper, published in “Frontiers” 1978, an appropriate name but a journal difficult to find. The main results of this group can be summarized as follows: (1) A time‐dependent inward current is recorded when hyperpolarizing clamps are applied from the maximum diastolic potential and fails to reverse at levels up to −160 mV (Fig. 6A and B). (2)Membrane conductance during the course of the inward current increased (Fig. 6C). (3) Rate of K^+^ depletion during pacemaker current is constant.

The lack of a reversal potential around the equilibrium potential for K^+^ ions suggests a pacemaker current by an ionic species with a positive reversal potential. The decrease in K^+^ conductance during pacemaker depolarization was considered too small to generate the diastolic depolarization. According to Hilary Brown's appreciation of the problems involved and their historical evolution and evaluation, Morad's group was the first to suggest activation of an inward current with a large positive equilibrium potential as the cause of the pacemaker depolarization. In her Physiological Reviews text, Brown (1982, p. 514) quotes the conclusion words of Maylie et al.: “These results do not support the turn‐off of a K^+^ conductance as the primary mechanism for the generation of the pacemaker potential in the SA nodal tissue; rather the results are more consistent with the idea that activation of an inward current, with large positive equilibrium potential, is responsible for pacemaking activity” and Hilary's final comment: “Indeed they were the first to make this suggestion.”

## What's in a Name? A New Interpretation (DiFrancesco 1981)

The end of the 1970s is remarkable and will be remembered among cardiac electrophysiologists as the period in which the outward current IK2, a “pure” K^+^ current, activated on depolarization disappeared from the scene and was replaced by If, a funny inward current, carried by Na^+^ and K^+^ and activated by hyperpolarization. It was the fall of IK2 and the rise of If as DiFrancesco remarked. Observations such as the disappearance of the IK2 current in Na^+^‐free medium (Deck and Trautwein 1964; McAllister and Noble 1966), and the reversal potential for IK2 at too negative potentials (Noble and Tsien 1968; Peper and Trautwein 1969) prepared the electrophysiological community for a change. The possibility to perform voltage clamp experiments on SAN preparations which showed an inward current activated on hyperpolarization weakened the IK2 type of explanation.

**Figure 7:**
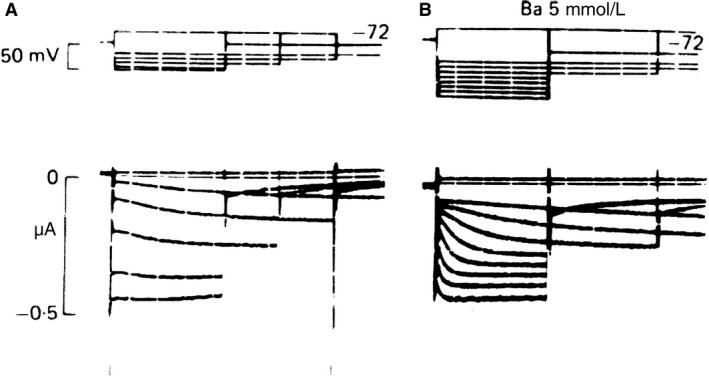
Reinterpretation of IK2. Voltage clamp on calf Purkinje fiber. (A) Hyperpolarizing in normal Tyrode reveals an apparent current reversal near −127 mV. (B): In the presence of 5 mmol/L Ba^2+^, no reversal is observed on hyperpolarizations in the range of −52 to −165 mV (DiFrancesco 1981). With permission.

**Figure 8:**
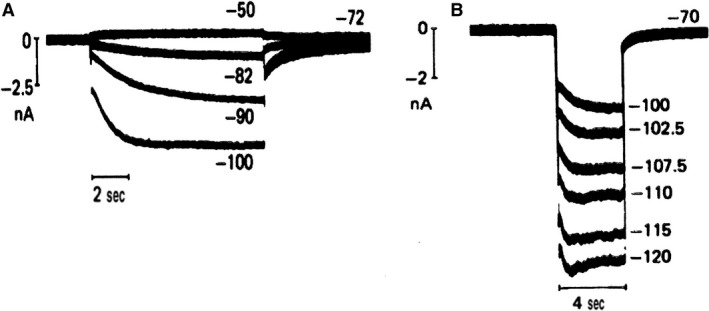
Confirmation of the existence of an increasing inward current upon hyperpolarization in sheep (A) and cow (B) single Purkinje cell. Absence of Ba^2+^ ions. Superimposed membrane currents during hyperpolarizing and depolarizing clamps in single Purkinje cells superfused with normal Tyrode solution containing 5.4 mmol/L K at 37°C. The numbers indicate the corresponding clamp potential in mV (Callewaert et al. 1984). With permission.

In January 1980, Dario DiFrancesco was ready to move. In a telephone call to Denis Noble, he announced that their interpretation of the IK2 story was not correct. He was convinced that the currents seen during a diastolic voltage clamp in Purkinje fibers were not dictated by the deactivation of an outward IK2 current but generated by the simultaneous change in two currents: an artificial IK1 depletion current and an increasing inward If current; Denis, as he recalls, made during that night a number of calculations with the program Dario and Denis had conceived and were reassured. So in the next weeks all kinds of control tests were executed: permeability for Na^+^and K^+^, change in reversal potential, kinetics. The crucial experiment that forced Dario to phone Denis was the use of Ba^2+^ ions (Fig 7). Ba^2+^ ions block the IK1 current and thus eliminate the depletion phenomenon responsible for the deformation of the hyperpolarizing current during a voltage clamp experiment. As evident from the figure, the reversal potential was absent and only an inward current increasing in time and amplitude remained. The If current in Purkinje fibers was born. Our Leuven laboratory (Callewaert et al. 1984) (Fig. 8) was happy to contribute a confirmation by showing in single Purkinje cells the presence of If. This observation was made in the absence of Ba^2+^ ions but in a preparation (single cell) without narrow clefts and hopefully not subject to depletion‐accumulation problems, certainly not in a major form. Noble and DiFrancesco found that our findings were **“**amply”(Noble 1984) and “straightforward” (DiFrancesco 1985). In the “surprising heart”, Noble (1984, p. 10) expressed the uneasiness created by the so‐called abrupt change in policy about the pacemaker current. He writes: “So my laboratory had been living already for 3 years with the tensions generated by the uncanny resemblance of the nodal If and IK2 and with the increasing theoretical difficulties posed by the IK2 model itself. … I had in fact with some rough calculations in hand concluded with Dick Tsien that there were not enough ions in the extracellular space to carry all the current that flows negative to the supposed reversal potential without massive replacement by diffusion.” The experimental results were not wrong, it was the interpretation that caused some trouble.

The discovery of If in the SAN brought a nice solution to the objections raised against the IK2 explanation for the Purkinje fiber but the If current was not directly or generally accepted as playing an important pacemaker current. Arguments against If's involvement were its too negative threshold and too slow kinetics. On the other hand, one should realize that experimental conditions may exert important effects: an increase in sympathetic tone or in intracellular Ca^2+^ concentration (Hagiwara and Irisawa 1989) for instance have shown to modify the activation voltage range and the amplitude of the If current (Callewaert et al. 1984). The discussion on the relevance of If for pacemaking is still going on. A demonstration thereof was again given on a meeting in Pécs 2017 with heavy discussions during a session on pacemaker activity.

## Is If The Only Pacemaker Current? Are More Currents Than If Involved?

The L‐type Ca^2+^ current is generally accepted to be responsible for the upstroke of the sinoatrial action potential. This is correct for the center of the sinus node. Around the turn of the century however, it has been discovered that in the periphery of the node, TTX slows the final part of the slow diastolic depolarization and the upstroke (Kodama et al. 1997). Two types of Na^+^ channels have been found to be expressed: Na^+^ channels of the neuronal type, sensitive to nanomolar concentrations of TTX and channels of the cardiac type sensitive to micromolar TTX concentrations (Lei et al. 2004).

Although IK2 was removed from the scene, this did not mean that other K^+^ currents (IK, Ix) were excluded from playing a role. Their deactivation in combination with an inward background current could still participate in causing the early diastolic depolarization. The following ionic currents have been proposed to carry inward current during the slow diastolic depolarization: Isustained, ICaT and INCX. The sustained inward current (Guo et al. 1995) is carried by positive cations, it is voltage‐activated, weakly inactivated, blocked by Mg^2+^ and Ca^2+^ channel blockers, stimulated by isoprenaline, resistant to TTX.

**Figure 9:**
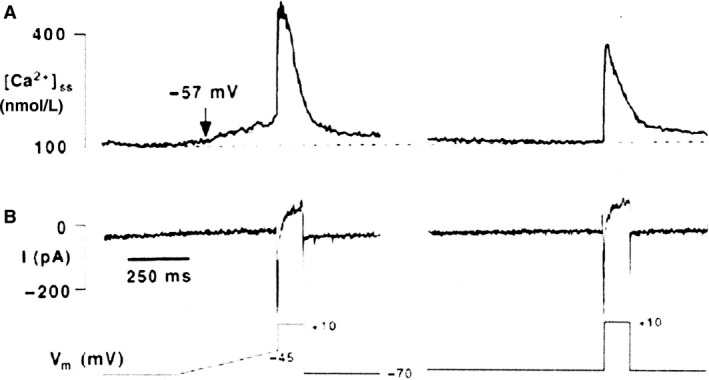
Pacemaker Ca^2+^ sparks are triggered by voltage‐dependent mechanism. Changes in subsarcolemmal Ca^2+^ (A) and membrane currents (B) in a latent atrial pacemaker cell in response to a depolarizing ramp clamp from −70 mV to −45 mV (400 msec) prior to the voltage step to +10 mV (150 msec). Low voltage Ca^2+^ release sparks were elicited. In the same cell without voltage ramp no sparks were detected (Hüser et al. 2000). With permission.

Apart from the L‐type Ca^2+^ current a second calcium current, the ICaT (T for transient) current has been described in atrial (Bean 1985) and ventricular (Nilius et al. 1985) cardiac cells, and later in SAN rabbit (Hagiwara et al. 1988). The threshold for activation of ICaT is negative to that of the L‐type Ca^2+^ current, the range of activation and potentials during the diastolic depolarization overlap. This makes it a good candidate to play a role in pacemaking. Its expression density in the SAN furthermore was found to be five times the level in atrial or ventricular cells (Hagiwara et al. 1988; Zhou and Lipsius 1994). It is blocked by Ni^2+^, but is not affected by adrenaline. In the presence of Ni^2+^, the late phase of the diastolic depolarization is markedly slowed. The role of T‐type Ca^2+^ current is however broader than providing a direct inflow of positive charge. It has been shown by Hüser et al. (2000) that Ca^2+^ release occurs during the late third of the diastolic depolarization, which suggests that the T‐type Ca^2+^current could be responsible as a trigger current (Fig. 9). The Ca^2+^ release in turn is paralleled by a slow inward current carried by INCX. The INCX as well as the Ca^2+^ release are blocked (indirectly) by Ni^2+^.

This brings us to the following sequence. At the beginning of the diastole, the membrane is depolarized as a consequence of IK deactivation and If activation; this leads to activation of the T‐type Ca^2+^ current, Ca^2+^‐induced Ca^2+^ release from the SR, and activation of INCX (Hüser et al. 2000) with further depolarization of the membrane till threshold of the L‐type Ca^2+^current or the Na^+^ current (in the periphery of the node) is reached. The system is redundant.

**Figure 10:**
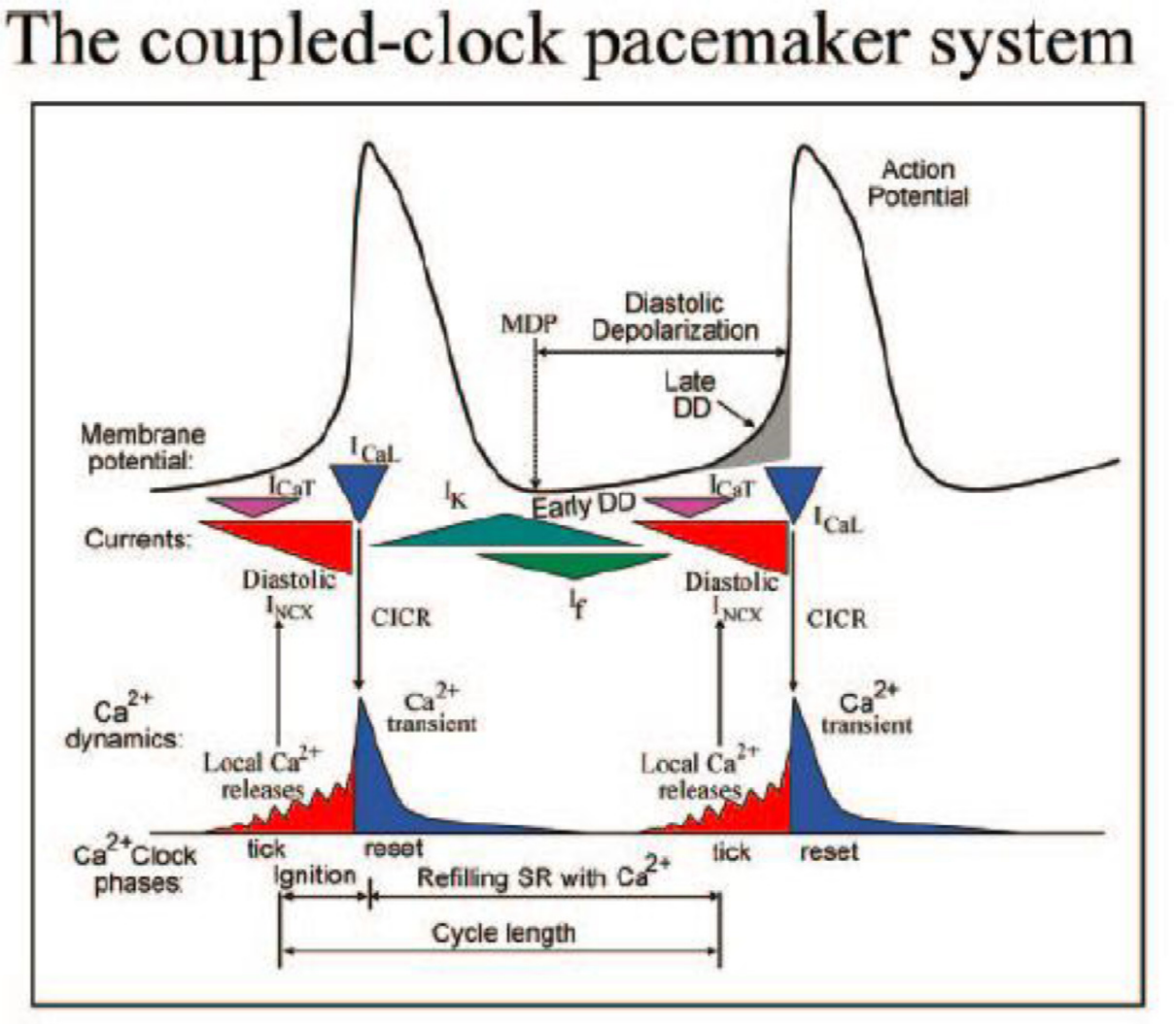
Schematic representation of the interaction between the membrane clock (membrane potential and ionic currents) and the calcium clock (Ca^2+^transients) (Lakatta et al. 2010). With permission.

According to the Lakatta's group (Lakatta et al. 2010), the release of Ca^2+^ also occurs spontaneously under the form of sparks or openings of single RYR‐sensitive Ca^2+^ channels, independently from a T‐type Ca^2+^current (Fig. 10). It can thus act as a separate mechanism. The T‐type Ca^2+^ current as a trigger is not required, which emphasizes the role of Ca^2+^ release as a primary clock mechanism. In this way, a distinction is made between the internal clock and the membrane clock. Both clocks interfere and both are modulated by voltage, subsarcolemmal Ca^2+^, PKA, Ca‐MK, phosphorylation, and G‐protein receptors (Fig. 10). The final performer is always the membrane clock which is not restricted to If. From the preceding analysis, it is evident that the rhythmic activity in the sinus node is not due to a single current but caused by an entity of different currents. To finish I may cite Noble et al. (2010) in “Competing oscillators in cardiac pacemaking”: “Already, earliest modeling and experimental work showed, consistent with current findings, that neither If nor spontaneous Ca^2+^ release from the SR on its own can be driving pacemaker activity. There is always concerted action necessary and interplay between the various ion channels.”

Presently, research in the field of genetic modulation has been limited to the If current and construction of mice models which are not always easy to interpret. Four isoforms of the HCN gene have been found to be expressed in cardiac tissue. In the SAN, the HCN4 (hyperpolarized cyclic nucleotide activated gene) has the highest expression. HCN3 has not been detected in the SAN (Bucchi et al. 2012) but is present in ventricular myocytes. It is important to mention that the role of HCN in heart is not restricted to sinoatrial cells. In some ventricular cells of hypertensive rats, diastolic depolarization and prolonged action potential have been observed (Cerbai et al. 1994). In these cells, a pronounced If current activated in the physiological range of membrane potentials was present. The If could thus be involved in the genesis of spontaneous activity in ventricular cells. Less directly expected was the observation of a pronounced prolongation of the action potential duration. Whether this is due to slow deactivation of If is still an open question. Experiments on preparations showing a normal positive, long plateau, and computer simulation seems required.

## Future Developments. Biological Pacemaker

Many cardiac patients survive thanks to the existence of electronic pacemakers. And yet there is a movement among electrophysiologists to substitute electronic pacemakers by biological pacemakers. Justification of this movement is the persistence of drawbacks still present in the available electronic pacing instruments.

Two types of approaches are being used in the development of biological pacing: the gene‐based methods and the cell‐based methods. A recent interesting development in the latter category is the use of transcription factor TBX18 to affect native cells in the receiving heart directly (i.e., without going through the stage of pluripotent cells in vitro) and transform them into pacemaker cells with a minimally invasive intervention. Within a few days, ventricular myocytes transformed into SAN cells (Kapoor et al. 2013). Experiments were performed in vitro on rat ventricular myocytes and in vivo on guinea‐pig hearts. TBX18 induced voltage clock and calcium clock automatism, sensitive to autonomic regulation; diastolic depolarization was dependent on HCN4 induced If current, and IK1 was reduced by 78%, a typical pacemaker behavior. The change in morphologic and electrophysiologic phenotype persisted beyond the Tbx18 expression. Along the same line, also fibroblasts have been transformed in cardiomyocytes (Ieda et al. 2010; Bakker et al. 2012).

Another method to produce a biological pacemaker, investigated by many groups, is to start from induced pluripotent mesenchymal stem cells (IPSC).

Using this approach**,** Protze et al. (2017) according to a recent study were able to generate SAN‐like pacemaker cells. When transplanted into the apex of rat hearts these cells were able to pace the host tissue. Pacemaker function of iPSC‐derived cardiomyocytes was also obtained in a canine model by M. Rosen and his group (Chauveau et al. 2017). Embryoid bodies were derived from human keratinocytes, and injected subepicardially into the left ventricle. After 4–13 weeks, the heart was removed and manifested If‐dependent automaticity. The authors conclude that iPSC‐CMs can integrate into the host myocardium and create a biological pacemaker. Although this is a promising development, rate and rhythm of the iPSC‐CMs pacemakers remain to be optimized.

These examples show that interesting openings have been made. Progress is slow but the step for the test in the clinic is approaching.

## Conflict of Interest

None declared.
